# Assessment of adherence to the National Malaria Guidelines in treatment management of malaria among children under five in integrated community case management in the DRC

**DOI:** 10.1080/16549716.2025.2545628

**Published:** 2025-08-29

**Authors:** Sahra Ibrahimi, Leon Kadiobo, Narcisse Embeke, Houleymata Diarra, Godéfroid Tshiswaka, Eric Mukumena, Bettina Brunner

**Affiliations:** a Department of Global Health, Denison University, Granville, OH, USA; b Global Development Group, Abt Global, Rockville, MD, USA; c President’s Malaria Initiative, USAID, Kinshasa, DRC; d National Malaria Control Program, Ministry of Health, Kinshasa, DRC

**Keywords:** Malaria, USAID IHP, the Democratic Republic of the Congo, community based program, primary data

## Abstract

**Background:**

Despite improved access to malaria healthcare, adherence to the National Malaria Guidelines for maintaining quality of care remains a concern in the DRC.

**Objective:**

We aimed to assess whether the management of malaria cases for children under 5 years of age using Integrated Community Case Management (ICCM) is conducted according to the National Malaria Guidelines in the DRC.

**Methods:**

We used a sample of 2,326 children from 30 ICCM sites. To determine adherence, we compared treatments received with items recommended by the National Malaria Guidelines. The chi-square tests were used to assess adherence to guidelines. Multiple logistic regression with adjusted odds ratios (AOR) and 95% confidence intervals (CI) were used to assess the association between the provinces and adherence to guidelines.

**Results:**

About 63.8% of children had malaria, and 94.8% (*n* = 1,407) of children who were clinically diagnosed with malaria received rapid diagnostic test (RDT) vs. 2.8% (*n* = 42) children who did not receive RDT, *p*-value = 0.0001. Additionally, those who had malaria were more likely to receive malaria treatment (93.4% vs. 6.6%, *p*-value = 0.0001). However, 18.5% of children who did not have malaria still received malaria treatment (*p*-value = 0.0001). Compared to Lualaba, ICCM sites in Kasai Oriental were more likely to adhere to RDT testing (AOR = 1.89, CI: 1.51–2.37) and Tanganyika was less likely to adhere to RDT testing (AOR = 0.40, CI: 0.81–0.92).

**Conclusion:**

Our study provides insight into ICCM’s compliance with the malaria guidelines in the DRC, which can inform programs and contribute to improving adherence to guidelines and the quality of care.

## Paper Context

**Main findings**: Our study indicates that adherence to the National Malaria Guidelines is generally 80% or higher regarding rapid diagnostic tests (RDT) testing and treatment, particularly among children with malaria in the DRC. Nonetheless, there is room for improvement in adherence to more accurate diagnoses, timely follow-up procedures, and the availability of RDT, especially in the province of Lualaba.

**Added knowledge**: This is the first study to shed light on adherence to the National Malaria Guidelines in the treatment management of malaria among children after the establishment of Integrated Community Case Management sites in the DRC.

**Global health impact for policy and action**: The findings of this study can help the country and policymakers take the necessary steps to build the capacity of health sites, improve adherence, and enhance the quality of care. This study may also inform future programs and policies in other countries with similar contexts.

## Background

Malaria is a disease caused by the bite of a female *Anopheles* mosquito, which carries *Plasmodium* parasites [1]. The most recent World Malaria Report indicates that in 2023, there were approximately 263 million malaria cases worldwide, an increase from 252 million cases reported in 2022 [2]. Malaria-related deaths were estimated at around 597,000 in 2023, slightly lower than the 600,000 deaths recorded in the previous year [2]. The WHO African Region remains disproportionately affected by malaria, accounting for about 94% of global cases and 95% of malaria deaths in 2023 [2]. Among these deaths, children under the age of five represented roughly 76% of the fatalities within the region [2]. More than half of the malaria deaths in the African Region were concentrated in just four countries: Nigeria (30.9%), the Democratic Republic of the Congo (11.3%), Niger (5.9%), and the United Republic of Tanzania (4.3%) [2].

Factors associated with malaria morbidity and mortality among children in the DRC include low adherence to the National Malaria Guidelines, inadequate quality of care [3,4], and limited availability of quality medicines [5]. The latter refers both to the circulation of substandard and falsified antimalarial drugs and to intermittent shortages or limited access to recommended first-line treatments, such as ACTs – particularly in pediatric formulations [5]. Additional contributing factors include limited differential diagnoses on the provider side, low socioeconomic status, delay in seeking medical care [6], self-medication, and lack of education on the children’s family side [7]. Among the mentioned factors associated with malaria death, accessibility and quality of care is one of the most prominent issues in the DRC [5]. Numerous patients have to travel more than 5 km to access a health facility in the country, and several health zones have low quality of care for the management of malaria cases [8,9]. Reasons cited for low quality of care include poor adherence to the National Malaria Guidelines, low availability of medical devices and medicines, and poor management of health centers. A previous observational study conducted in the DRC between 2018 and 2020 showed only 50.3% of patients under five received parenteral antimalarial medication and an oral artemisinin-based combination therapy (ACT) [5]. In ICCM settings, where this study is situated, the focus is on early detection and treatment of uncomplicated malaria through RDT testing and ACT administration by community health workers. Severe malaria cases, in contrast, require parenteral treatment and urgent referral, which present different logistical and resource challenges.

Several donor organizations, including the Global Fund, U.S. President’s Malaria Initiative, Against Malaria Foundation, and United Nations International Children’s Emergency Fund (UNICEF) have supported various programs to address the issues of accessibility and quality of care and reduce the rate of malaria mortality in the DRC [10–14]. For example, since 2012, the World Health Organization (WHO) and UNICEF have developed the Integrated Management of Newborn and Child Illnesses (IMCI) strategy, which includes ensuring the comprehensiveness, effectiveness, and continuity of care and to reduce malaria deaths, among other diseases [11]. The DRC Ministry of Health has adopted the IMCI strategy in integrated community case management (ICCM) sites to meet the healthcare needs of the population; however, the DRC still faces challenges in adhering to IMCI protocols and making quality healthcare accessible to its population [5].

### Intervention and study objectives

In 2022, the Ministry of Public Health, Hygiene and Prevention, with support from the USAID Integrated Health Program (IHP) program and other partners, established more than 10 thousand sites, including new and existing ones, across 26 provinces. Of these 10 thousand sites, 3,400 were supported by USAID IHP in the nine project-supported provinces (Kasai Oriental, Kasai Central, Sankuru, Lomami, Sud Kivu, Haut Lomami, Haut Katanga, Lualaba, Tanganyika) [15]. About 291,268 children under 5 years of age were seen for a set of three childhood illnesses, including 184,392 cases of malaria, over the period from October 2020 to September 2021 in the 3,400 ICCM sites supported by USAID IHP. The establishment of sites has improved access to health services and the management of illness cases in children under 5-years old.

However, adherence to the National Malaria Guidelines for maintaining quality of care remains a concern in the DRC. The National Malaria Guidelines are actionable and evidence-based recommendations by national and international experts for malaria diagnosis and treatment of the population, including children under five [16]. The guidelines also provide recommendations about comorbidities (e.g. screening and referral of suspected cases of tuberculosis and HIV to healthcare institutions) and different treatment modalities such as community health worker treatment, pre-referral treatment, and treatment in an epidemic [16]. Specific malaria guidelines for children under five include proper and validated multispecies rapid diagnosis tests (RDT), timely follow-up (on the third day after the start of treatment), and appropriate treatment, among others.

The objective of this study is to assess whether the management of malaria cases for children under 5-years old in community care sites, implemented by USAID IHP, is conducted according to the National Malaria Guidelines. To determine adherence, we compare treatments received to what is recommended by the National Malaria Guidelines. Our study is essential for identifying challenges and opportunities in adherence to malaria guidelines and maintaining quality care in the DRC.

## Methods

### Data source and sampling

The data contained all patients under five who visited ICCM sites with symptoms suggestive of malaria during 3 months from October to December 2023 among children under 5 years old (*n* = 2,331). Malaria prevalence is usually highest during the rainy season, which generally spans from October to May, with peak transmission occurring between December and April. This suggests that our study may have captured more cases of malaria than usual. A multi-stage cluster convenience sampling was used to ensure that the sample size is large enough to be representative of the larger population. First, three provinces (Lualaba, Kasai Oriental, and Tanganyika) were selected, based on their higher number of functional ICCM sites. In the second stage, two health zones per province were selected, for a total of six health zones. Finally, at the third stage, 10 ICCM sites were identified and selected in each province, which was 5 ICCM sites per health zones, totaling in 30 ICCM sites.

The medical health record sheets from the health zones were collected manually by community healthcare workers at ICCM sites, and then data managers transferred the data electronically to the IASO (a cloud-based secure backup server for data). Data collectors were trained, and data confidentiality was ensured through deidentifying the data. The 2,331 medical health record sheets included patients’ sociodemographic and health status data, such as age, sex, vaccine status, well-child visit, reasons for visiting the health facility, as well as information on diagnoses and testing (e.g. RDT testing), treatment, and follow-up by the healthcare providers at the health zones. To minimize data reporting bias, we sampled only from ICCM sites that we knew had a functional data recording and reporting system. Additionally, we restricted our sample size to 2,326 children who had data on all variables of interest. We received institutional review board (IRB) exemption from Abt Global IRB on 1 November 2023, given that we are using deidentified secondary data.

### Data measurement and analysis

Adherence to guidelines was measured by assessing the following factors: whether patients received an RDT test if they had fever, diarrhea, and other symptoms of malaria; if they tested positive, whether they received treatment; if tested negative, whether they still received malaria treatment; whether patients had a follow-up visit; and whether community health workers checked to see if patients had taken the medication as prescribed. The above binary variables were defined as ‘yes’ (coded as 1) if guidelines were adhered to and ‘no’ (coded as 0) if they were not adhered to within each respective category.

Bivariate analysis was used to describe participant characteristics. The Chi-square test was also used to assess adherence to guidelines, with a value below 0.05 considered statistically significant. Finally, logistic regression was used to assess whether adherence (vs. non-adherence) to guidelines is associated with province of residence among patients. In this model, we controlled for demographics, including age, sex, vaccine status, well-child visit (also known as childcare visit), and home treatments. The results are presented as adjusted odds ratios with 95% confidence intervals, and all tests of hypothesis were two-tailed with a type 1 error rate set at 5%. Additionally, the complex survey sample design was accounted for using the svy Stata command. We used Stata 17 for data analysis.

## Results

Among the 2,326 children under five, the mean age was 29 months with a range of 1 month to 5 years old and standard deviation of 18, and about 51% (*n* = 1,191) of them were females (Table 1). Most of the children were from Tanganyika province (53.4%, *n* = 1,243), followed by Kasai Oriental (32.7%, *n* = 761), and Lualaba (13.8%, *n* = 322). The majority of children had previously received their required vaccination series (80%) and well-child visit (78.8%), while some children also received home treatment for malaria (20.9%). Additionally, most of the children visited the community care sites because of fever complaints (48.4%) and the rest were for other reasons such as diarrhea and cough or congestion. The mentioned proportion of health records on vaccination, well-child visit, home treatment, and health complaints were similar across the three provinces.

**Table 1.** Characteristics of sample participants in the DRC, 2024. *N* = 2,326.

Variables	Total, n (%)		Kasai Oriental n (%)		Lualaba n (%)		Tanganyika n (%)		*p*-Value
	2,326 (100%)		761 (32.7)		322 (13.8)		1,243 (53.4)		
**Age (months)**									<0.0001
Mean (SD)	29	(18.0)	30.0	(20.2)	33.8	(18.8)	27.1	(16.0)	
**Sex at birth**									<0.971
Male	1,135	(48.8)	374	(49.1)	157	(48.8)	604	(48.6)	
Female	1,191	(51.2)	387	(50.9)	165	(51.2)	639	(51.4)	
**Vaccine status**									<0.0001
No	466	(20.0)	103	(13.5)	55	(17.1)	308	(24.8)	
Yes	1,860	(80.0)	658	(86.5)	267	(82.9)	935	(75.2)	
**Well-child visit**									<0.006
No	494	(21.2)	137	(18.0)	62	(19.3)	295	(23.7)	
Yes	1,832	(78.8)	624	(82.0)	260	(80.0)	948	(76.3)	
**Home treatment**									<0.0001
No	1,841	(79.1)	668	(87.8)	257	(79.8)	916	(73.7)	
Yes	485	(20.9)	93	(12.2)	65	(20.2)	327	(26.3)	
**Reason for visiting**									<0.0001
Fever	1,116	(48.4)	466	(62.1)	159	(50.2)	491	(39.7)	
Diarrhea	348	(15.1)	111	(14.8)	87	(27.4)	150	(12.1)	
Cough/congestion	768	(33.3)	152	(20.3)	54	(17.0)	562	(45.4)	
Other	72	(3.1)	21	(2.8)	17	(5.4)	34	(2.7)	
**Received RDT**									<0.0001
No	157	(6.8)	32	(4.2)	13	(4.0)	112	(9.0)	
Yes	1,892	(81.3)	667	(87.7)	144	(44.7)	1,081	(87.0)	
RDT not available	277	(11.9)	62	(8.2)	165	(51.2)	50	(4.0)	
RDT result*									<0.0001
Malaria positive	1,407	(74.4)	471	(70.6)	113	(78.5)	823	(76.1)	
Malaria negative	485	(25.6)	196	(29.4)	31	(21.5)	258	(23.9)	
Received malaria treatment*									<0.0001
No	784	(33.7)	274	(36.0)	132	(41.0)	378	(30.4)	
Yes	1,542	(66.3)	487	(64.0)	190	(59.0)	865	(69.6)	

*Footnote: The sample size for RDT result and treatment received are restricted to only those who received an RDT test.

Overall, 81.3% (*n* = 1,892) of patients with symptoms of malaria including fever, received RDT testing, while 11.9% (*n* = 277) did not receive testing because an RDT was not available at the time of patients’ visit. Unavailability of RDT tests was highest in Lualaba (51.2%) compared to Tanganyika (4.0%) and Kasai Oriental (8.2%). Among patients who had an RDT result, 74.4% of children tested positive for malaria, with the highest proportion of malaria-positive children in Lualaba Province (78.5) followed by Tanganyika (76.1%), and Kasai Oriental (70.6%).

Table 2 presents adherence to the National Malaria Guidelines. Compared to children without malaria, 94.8% (*n* = 1,407) children clinically diagnosed with malaria received RDT testing vs. 2.8% (*n* = 42) children who did not receive RDT testing, including due to unavailability (2.4%, *n* = 35), *p*-value = 0.0001. Additionally, those who had malaria were more likely to receive malaria treatment (93.4% vs. 6.6%, *p*-value = 0.0001). However, 18.5% of children who did not have malaria still received malaria treatment and this association was also significant (*p*-value = 0.0001). In terms of checking in with patients to verify whether they are taking the medication prescribed, only 63.8% children with malaria were confirmed to have been taking the first dose of the medication at the time of the first visit by the community health worker. Patients with malaria were more likely to have a check in about taking medication (63.7% vs. 26.7, *p*-value = 0.001) compared to those without malaria.

**Table 2.** Adherence to guidelines in the DRC, 2024. *N* = 2,326.

Variables	Total, n (%)		Malaria Status				*p*-Value
	2,326 (100%)		No, 842 (36.2%)		Yes, 1,484 (63.8%)		
**Patients received RDT**							<0.0001
No	157	(6.8)	115	(13.7)	42	(2.8)	
Yes	1,892	(81.3)	485	(57.6)	1,407	(94.8)	
RDT not available	277	(11.9)	242	(28.7)	35	(2.4)	
**Received malaria treatment**							<0.0001
No	784	(33.7)	686	(81.5)	98	(6.6)	
Yes	1,542	(66.3)	156	(18.5)	1,386	(93.4)	
**Verified whether the child took the medication at the time of the visit**							<0.001
No	582	(25.0)	186	(22.1)	396	(26.7)	
Yes	1,485	(63.8)	539	(64.0)	946	(63.7)	
Patients had a follow-up*							<0.0001
No	2,130	(91.6)	720	(85.5)	1,410	(95.0)	
Yes	196	(8.4)	122	(14.5)	74	(5.0)	
New cases of malaria among returning patients*							<0.0001
No	1,996	(85.8)	717	(85.2)	1,279	(86.2)	
Yes	86	(3.7)	7	(0.8)	79	(5.3)	

*Footnote: Data missingness was 11.1% for medication status variable and 10.5% for new cases of malaria at the follow-up.

Regarding follow-up appointments, 8.4% (*n* = 196) of the patients had a follow-up, of which 74 had malaria. Patients who had malaria were less likely to have a follow-up (5% vs. 95%, *p*-value = 0.0001) compared to patients without malaria. Lastly, post follow-up, about 3.7% (*n* = 86) patients returned to the health facility with persisting symptoms of malaria of which 79 had malaria. Those who had malaria were less likely to be new cases of malaria (5.3% vs. 86.2%, *p*-value = 0.0001).

Table 3 presents results from the multiple logistic regression and includes adjusted odds ratios (AOR) with 95% confidence intervals for the association between provinces and adherence to guidelines among children with malaria (*n* = 1,472). Compared to Lualaba, Kasai Oriental was more likely to adhere to RDT testing (AOR = 1.89, CI: 1.51–2.37) and Tanganyika was less likely to adhere to RDT testing (AOR = 0.40, CI: 0.81–0.92). Additionally, compared to Lualaba, both Kasai Oriental (AOR = 0.75, CI: 0.62–0.91) and Tanganyika (AOR = 0.55, CI: 0.41–0.74) were less likely to adhere to treatment. However, compared to Lualaba, both Kasai Oriental (AOR = 2.95, CI: 2.64–3.29) and Tanganyika (AOR = 4.21, CI: 3.66–4.84) were more likely to verify that the child took medication.

**Table 3.** Multiple logistic regression models indicating adjusted odds ratios with [95% confidence intervals] for the association between province and adherence to guidelines among children with malaria in the DRC, 2024. *N* = 1472.

Provinces	Adherence		
	RDT testing	Treatment	Verified the child took medication
Lualaba	Ref.	Ref.	Ref.
Kasai Oriental	1.89 (1.51–2.37)**	0.75 (0.62–0.91)*	2.95 (2.64–3.29)**
Tanganyika	0.40 (0.18–0.92)*	0.55 (0.41–0.74)*	4.21 (3.66–4.84)

*Footnotes*: Significance: <0.05*, 0.00**, 0.000***.

## Discussion and implications

Our findings indicate that overall adherence to the National Malaria Guidelines is 80% or higher concerning RDT testing and treatment. Adherence is particularly strong among children with malaria (94.8% for testing and 93.4% for treatment). However, adherence to more accurate diagnosis and follow-up, including confirming children’s medication intake, requires further improvement. For example, 18.5% of children without malaria still received malaria treatment, which is contrary to guideline requirements specifying treatment only for those with confirmed positive results. This finding is consistent with previous studies conducted in sub-Saharan countries that found low adherence to malaria guidelines set by their ministries of health or the World Health Organization (WHO) [5,17–19]. Reduced adherence to National Malaria Guidelines may be due to providers’ insufficient training and compliance with the guidelines. A study in Kenya showed that none of the health facilities had a copy of the National Malaria Guidelines and only staff in 20% of the facilities adhered to the guidelines [20]. The study also found that the likelihood of adherence to guidelines was higher among more educated and trained staff [20].

Furthermore, while the guidelines require all individuals suspected of having malaria to undergo testing, we found that 277 patients with suspected cases of malaria did not receive RDT testing due to the unavailability of RDTs at the time of the patients’ visit. Such shortages in supply and lack of availability of testing or medication may adversely impact adherence to guidelines. A study in Ethiopia showed that one of the determinants of reduced adherence of providers to malaria guidelines was interruptions in the supply of laboratory equipment, including testing and antimalarial medications [17]. Health facilities must communicate supply shortages and other facility issues with the ministry of health and other aid organizations in a timely manner. Providers should also have extra supplies in their health facilities for unforeseen situations.

Additionally, compared to the 51.2% lack of testing for suspected cases due to unavailability of RDT in Lualaba, the lack of testing for suspected cases due to unavailability of RDT was only 8.2% in Kasai Oriental and 4.0% in Tanganyika. Ensuring the availability of RDT testing and correct conduct of RDT diagnostic testing for suspected malaria cases is essential in managing malaria cases in the DRC. Therefore, merely establishing malaria guidelines is not sufficient; RDT testing should be made available to all ICCMs, and healthcare professionals need to actively implement those guidelines. Moreover, adherence to RDT testing and malaria treatment differed among the provinces. For instance, compared to Lualaba, adherence to RDT testing was high in Kasai Oriental and low in Tanganyika. These findings about the provinces can help the DRC and aid programs to tailor their interventions and strengthen those provinces in most need of support.

While providers are considered responsible for adhering to guidelines at the stages of diagnosis, screening, and treatment, parents of the children also share the responsibility of complying with recommended dosing and bringing their children to the health facility when follow-up is needed. Previous studies in the DRC, Nigeria, and Uganda have shown that the required series of treatment was not completed due to lack of compliance and follow-up, which may lead to a significant risk of incomplete clearance of parasites and the resurgence of the disease [5]. Therefore, the importance of completing all series of medication and treatment should be emphasized to both the providers and families.

Lastly, improving adherence to National Malaria Guidelines requires comprehensive strategies beyond provider training and supply chain management. Ongoing educational programs should emphasize practical skills in diagnosis, dosing, and follow-up care, supported by routine monitoring and supervision to ensure consistent implementation [21]. Additionally, policymakers and program implementers must consider the broader political, economic, and social determinants that affect health system functionality. Issues such as political interference, corruption, and socioeconomic disparities significantly challenge malaria control efforts in the DRC and similar high-burden countries [22]. Addressing these requires multisectoral collaboration, transparency initiatives, and sustained investment in health infrastructure and governance [23]. Only through an integrated approach that combines operational improvements with systemic reforms can the quality of malaria care be substantially enhanced.

The findings of this study can inform program implementers and policymakers in areas for improving quality of malaria care for children in the DRC. A strength of our study is the large and representative sample from three provinces. Additionally, the data was collected following the implementation of malaria programs by the USAID IHP in the DRC. Some limitations of this study include lack of data on providers’ level of education and training that may impact adherence to guidelines and patients’ health outcomes. Another limitation of this study is that the treatment received might have been slightly under- or over-estimated, as there was no systematic verification system in place. Since the data come from registers, some providers might have misreported a negative test as positive to warrant provision of an antimalarial. Data on whether community health workers confirmed medication intake rely on recorded observations, which may be subject to reporting bias, and actual adherence cannot be independently verified. Additionally, the study did not include data on providers’ assessment of severity signs, exact drug dosages administered, or observation of first doses, which limits our ability to fully evaluate the quality of malaria case management. Finally, this study did not include other vulnerable populations such as pregnant women; however, malaria occurrence among children is more prevalent. Thus, this research offers valuable understanding regarding challenges and opportunities in adherence to National Malaria Guidelines and improving the health of children under five in integrated community case management sites.

## Conclusion

Our study indicates that adherence to the National Malaria Guidelines is generally 80% or higher regarding RDT testing and treatment, particularly among children with malaria, in the DRC. Nonetheless, there is room for improvement in adherence to more accurate diagnoses and timely follow-up procedures, such as ensuring children are consistently taking their medication. It is not sufficient to have established guidelines about malaria; healthcare providers must put those guidelines into practice. Operationalizing the guidelines requires the intervening programs to assess whether community care sites have a copy of the guidelines and are receiving training on applying the guidelines and communicating any issues with the supervisor and aid organizations. Improved adherence to guidelines also requires sufficient provision of RDTs and ACTs. Our study shed light on the status of the integrated community case management compliance with the National Malaria Guidelines in the DRC, which can help the country take the necessary steps to improve adherence and the quality of care. This study may also inform future programs and policies in other countries with similar contexts. Future studies should consider a qualitative study conducting in-depth interviews with providers and parents of those children with malaria to better understand the reasons for non-compliance of the guidelines and inadequate quality of care.

## Data Availability

Data may be shared privately upon reasonable request.
